# Training and usage of detection dogs to better understand bumble bee nesting habitat: Challenges and opportunities

**DOI:** 10.1371/journal.pone.0249248

**Published:** 2021-05-12

**Authors:** Amanda R. Liczner, Victoria J. MacPhail, Deborah A. Woollett, Ngaio L. Richards, Sheila R. Colla

**Affiliations:** 1 Department of Biology, York University, Toronto, Ontario, Canada; 2 Faculty of Environmental and Urban Change, York University, Toronto, Ontario, Canada; 3 Working Dogs for Conservation, Missoula, Montana, United States of America; Ghent University, BELGIUM

## Abstract

Bumble bees are among the most imperiled pollinators. However, habitat use, especially nest site selection, remains relatively unknown. Methods to locate nests are invaluable to better understand habitat requirements and monitor wild populations. Building on prior study findings, we report constraints and possibilities observed while training detection dogs to locate bumble bee nests. Three conservation detection dogs were initially trained to three species of bumble bee nest material, first within glass jars concealed in a row of cinder blocks, then placed in the open or partially hidden for area searches. The next intended training step was to expose the dogs to natural nests located by community science volunteers. However, significant effort (> 250 hrs), yielded only two confirmed, natural nests suitable for dog training purposes. Although the dogs did not progress past the formative training stage valuable insight was gained. Maximum observed detection distance for bumble bee nest material during initial controlled training was 15 m, which decreased significantly (< 1 m) once training progressed to buried samples and natural nests. Three main considerations around future training and usage of detection dogs were identified. First, dogs might benefit from transitional training via exposures to known natural nests, regardless of species. However, it may be too difficult for people to find natural nests for this, and prior work demonstrated the ability of dogs to generalize and find natural nests after testing to artificially-buried nest material. Second, confirming a dog’s nest find, via resident bee presence, is nuanced. Third, future study design and objectives must harness strengths, and reflect limitations of detection dog surveys and search strategies, as extensively discussed in this paper. Prospective studies involving detection dogs for locating bumble bee nests would benefit from considering the drawbacks and opportunities discussed and can mitigate limitations through incorporating these considerations in their study design.

## Introduction

Approximately 25% of bumble bees assessed by the IUCN (International Union for Conservation of Nature https://www.iucnredlist.org) are in decline [1, 2] with habitat loss acknowledged as one of the main threats [2, 3]. Agricultural intensification and development have decreased floral resources [4, 5] and little is known about the loss of available nesting and overwintering sites through increasing impermeable surface cover, loss of grasslands and a reduction of forest cover. Permeable surfaces are needed for overwintering and nesting sites. Queens overwinter by burying themselves underground, often at the base of trees [6–8] and many bumble bee species also nest underground [9]. Conserving and ensuring the availability of viable bumble bee nesting habitat is important for mitigating declines in bumble bee populations.

Most bumble bee conservation efforts and habitat studies have focused on increasing floral resource availability [e.g. 10, 11] while nesting and overwintering resources have received comparatively little consideration. This may be because bumble bee nests are often well-concealed and thus difficult to locate [9]. The entrances to bumble bee nests are usually very small and typically only detected when bees are frequently observed entering/exiting an opening or openings [e.g. of multiple entrances 12, 13, S. O’Connor pers. comm. 2019]. Bumble bee habitat studies should focus on nesting requirements because it is an important determinant of a species’ critical habitat, a parameter which must be defined for legal protections, and locating bumble bee nests allows monitoring demographic responses of individual species for determining habitat quality [14]. To manage declining bumble bee species, it is important that we increase our knowledge of nesting habitat and our ability to locate nests.

Despite wild pollinator conservation being an active area of research, naturally-occurring bumble bee nests remain difficult to locate and there is an urgent need to develop approaches to increase our understanding of habitat selection. Previous studies investigating bumble bee nesting habitat have used various methods from inferential to observational to locate nests [9]. Examples of inferential strategies include: molecular methods to identify the number of colonies in a habitat [15], statistical modelling of foragers to predict nest location [16], and the presence of nest-searching queens as a proxy for nest locations [17]. These methods offer relatively quick and easy identification of the number of colonies within the landscape but often cannot locate the actual nest site. Without actual nest locations identified, valuable information including small scale habitat characteristics, ecological requirements, bee activity, and demographic information, will be missed. Observational methods usually involve slowly walking transects within a site to locate nests [18] or opportunistic sightings of bumble bee nests [19]. Although these methods are time and labour intensive and usually result in few nests being located without substantial effort, they do offer an advantage of directly finding bumble bee nests. This underscores the fact that there remains a need for efficiently and effectively locating bumble bee nests.

Potentially complementary approaches that could facilitate direct searches for bumble bee nests have been explored in pursuit of better describing nesting habitat preferences. Two previous studies in the United Kingdom assessed the viability of using dogs to locate bumble bee nests [20, 21]. Detection dogs have successfully been used to facilitate or optimize the finding of numerous wildlife species and targets deemed to be of high or urgent conservation value, or which help inform assessment of habitat quality and selection [e.g. 22 Table 10.1]. Detection dog-assisted searches of a location can often be conducted rapidly, while offering comprehensive survey coverage of an area [e.g. 23]. The use of detection dog-handler teams as a survey tool may also introduce less bias than human-only efforts, potentially leading to exploration and finds in unexpected areas that might not otherwise have been searched [e.g., river otter and American mink fecal matter along river banks, 24]. Similarly, the use of detection dogs may increase the likelihood of finding a target that is more difficult for humans to visually detect or discern [e.g., invasive/noxious weeds as part of latter stage eradication efforts 25].

As part of broader bumble bee conservation research, and building from prior work [20, 21], we had an objective of training and then fielding (in this case surveying in targeted areas to gather data on previously unencountered naturally-occurring bumble bee nests) conservation detection dog-handler teams in southern Ontario, Canada in the summer of 2019. Here, we attempted to apply a transitional (and previously found to be effective) training step [26], of offering the dogs multiple exposures to known naturally-occurring nests. We also examined previously reported parameters [e.g. find rate, 20, 21] and conferred with authors of prior bumble bee studies on unreported variables (e.g., detection distances; S. O’Connor, pers. comm. 2019). Although the specific objective of formally surveying with the dogs for naturally-occurring nests could not ultimately be fulfilled, new insight was acquired on whether, and how, to best proceed with this type of work. Herein we describe the primary challenges that the professionally-trained conservation detection dogs, and the humans tasked with searching for naturally-occurring nests in conjunction with those efforts, encountered during the process and discuss potential opportunities for further research going forward.

## Materials and methods

### Initial bumble bee nest sample acquisition and dog training

Trainer-handlers from a conservation dog organization (Working Dogs for Conservation; WD4C, Missoula, Montana) conducted initial dog training in a controlled environment (see below) in Montana, USA [26–29] (June 3 to 19, 2019), followed by *in-situ* training and exploratory ground-truthing near our designated study sites (Silver Creek Conservation Area (43.691917, -79.966178), and Terra Cotta Conservation Area (43.721493, -79.959306) in southern Ontario, Canada (June 24, to July 5, 2019). These field sites are protected areas for which we obtained permits prior to conducting field surveys from the Credit Valley Conservation Authority (permit number CL 19/007).

The training samples used in Montana were from seven nests of three bumble bee species (Table 1). Samples consisted of wax cells constructed by the bees (honey pots with and without nectar, brood cells) and some deceased worker bees. Nests were initiated by wild-caught queens that were reared in lab conditions at the University of Illinois. Samples were stored in a freezer at -20°C until shipment to WD4C in a Styrofoam cooler on ice packs. Upon arrival, they were separated into three distinct subsamples (to offer as training samples to the dogs), placed in fresh, labeled Ziploc^®^ bags (sandwich-size) and stored in a freezer at -20°C until training began. Once used for training each subsample was subsequently stored in sealed glass jars, further detailed below. Note that the use of such captive-reared nest material is consistent with the training the two dogs in prior work received to commercially-reared nests [i.e., 20, 21].

**Table 1:** A description of the training bumble bee nest samples the dogs were exposed to in Montana, USA and Ontario, Canada.

Training Location	Type of training	Species	Number of nests	Captive or Naturally-occurring nests	Source
**Montana**	Lineup and area search (placed in mowed grass, garden)	*B*. *impatiens* Cresson, 1863	4	Captive colony founded by wild-caught queens from Illinois	Dr. Benn Sadd, University of Illinois
**Montana**	Lineup and area search (placed in mowed grass, garden)	*B*. *terricola* Kirby, 1837	2	Captive colony founded by wild-caught queens from Vermont	Dr. Benn Sadd, University of Illinois
**Montana**	Lineup and area search (placed in mowed grass, garden)	*B*. *occidentalis* Greene, 1858	1	Captive colony founded by wild-caught queen from Oregon	Dr. Benn Sadd, University of Illinois
**Ontario**	Area search, placed (on ground, in vegetation, buried max 5 cm)	*B*. *impatiens* Cresson, 1863	2	Commercial colony	BioBest
**Ontario**	Area search	*B impatiens* Cresson, 1863/ *citrinus* (Smith, 1854)	1	Naturally-occurring colony from Ontario	Reported by private citizen, Norwich, Ontario
**Ontario**	Area search	*B*. *bimaculatus* Cresson, 1863	1	Naturally-occurring colony from Ontario	Reported by private citizen, Guelph Ontario.

Training nest samples were bits of nesting material separated from the rest of the nest (approximately 2 g of material) containing wax cells, honey pots, and brood cells. The training location is where the dogs were exposed to the nesting material, while source is where the nest training samples originates from.

Three conservation detection dogs (two male border collies and a female Belgian Malinois) received introductory exposure to bumble bee nest material, using standard and previously established conservation dog training methods [26–29]. The three dogs that participated in this study are owned by WD4C. The three trainer-handlers who also directly participated in the study ensured proper care and use of the dogs under the operational parameters of WD4C, which meet or exceed requirements of the United States Department of Agriculture (USDA) Animal Welfare Act and Animal Welfare Regulations Part 3, Subpart A, which addresses handling, care, treatment, and transportation of dogs.

All dogs had previous experience training to, and surveying for, other conservation detection targets prior to this study. A thorough consideration of the traits and characteristics that WD4C and numerous other conservation dog groups seek, regardless of breed, can be found in MacKay et al. [28]. Extensive details regarding training procedures and approaches can be found in [26–29], but briefly, during the initial sessions in Montana, the dogs were first presented with an array of cinder blocks within which a training sample was concealed in some of the blocks in a sealed, 8 oz glass canning jar (Ball ®, regular mason) with a perforated lid. Training then proceeded to dogs free searching off-leash within a fenced area (approximately 250 m^2^) for samples that were placed in low lying vegetation (mowed grass, garden). Approximately 2 g of nesting material was used in each training exposure. Each dog indicated recognition of the bumble bee nest material (i.e., the target scent) by giving a passive “alert” (i.e., sitting or lying beside it), for which they were rewarded through a play session with the handler. Each dog received 126 individual exposures to training nest samples from all three bumble bee species before departing for Ontario. The purpose of training conducted in Montana was for the dogs to gain familiarity to the scent of bumble bee nest materials and to generalize to multiple species. In this application, generalization refers to dogs being able to detect bumble bee nests of additional species not presented to them during initial training, i.e., progressing from exposure solely to commercially/captive-reared nest material in training to recognition of (previously unencountered) wild/naturally-occurring nest material once training in-situ. Whenever observed, the detection distances, from where the dogs exhibited a “change in behaviour” indicating they had picked up the scent relative to the placed sample, were noted. Other noteworthy elements (e.g., nest ID and subsample number used relative to training session) were also recorded at the different progressions reached during this initial phase of training.

### Human naturally-occurring nest searches for in-situ dog training

The bumble bee nest detection work previously undertaken with dogs [20, 21], and direct communication with one of the primary authors of those studies (S. O’Connor, pers. comm. 2019), revealed that dogs have the ability to generalize, (i.e., being trained using nest samples from certain bumble bee species and detecting placed nest material and naturally-occurring nests from other bumble bee species) both during field testing and once formally fielded (i.e., actively searching for nests). In parallel, WD4C trainer-handlers have found that even if/when dogs have shown an ability to generalize to a particular target, offering exposure opportunities to known/confirmed targets *in-situ* can be beneficial to dogs prior to fielding. As such, *in-situ* exposure has become an additional, warranted training process for many targets to which dogs have shown they can generalize, but which produce multiple scent variants and/or a smaller amount of scent. Therefore, while the dog team training was underway in Montana, a concerted attempt was being made in southern Ontario to locate naturally-occurring bumble bee nests so that the dogs could undergo the transitional training stage and be promptly reinforced for finding *in-situ* naturally-occurring nests that were not handled or placed by humans. Researchers and volunteers searched for nests within the designated study sites. A social media campaign was also launched to increase the chances of finding bumble bee nests across southern Ontario, particularly on private property.

ARL began searches for bumble bee nests on May 1, 2019 between 9:30 am to 3:30 pm until July 5. VJM, SRC and 17 community science volunteers also searched for bumble bee nests from June 17 until July 5. Searches were conducted two to three times per week on warm, sunny days from 10:00 am to 4:00 pm. Prior to beginning nest searches, all volunteers were instructed on how to identify a bumble bee from other bees, what bumble bee nesting behaviour could look like (orientation flights, queens with pollen entering a potential nest site, worker bees entering/exiting a potential nest site), common nesting locations [7, 30–32] and distinguishing between bumble bee foraging and nest-searching behaviour. Community scientists were not required to have any prior experience with field work or bumble bee identification. For the first two weeks, volunteer nest searches at the study site were conducted by slowly walking the trail system throughout the conservation areas searching on either side of the trail for signs of nesting activity. Previous studies looking for bumble bee nests slowly walked pre-designated transects through study sites [i.e., 18], however, we were required to stay on the established trail system as much as possible by the conservation authority as sites were within protected areas. Locations within the study areas with many nest-searching queens were noted as areas to concentrate nest searches as high nest-searching queen activity has been previously associated with bumble bee nest activity [33]. The second two weeks were spent searching targeted locations for between 30–90 minutes as the average foraging duration for workers (which we assumed to be similar for queens) has been measured for at least 31 minutes [34]. These target areas either had high nest-searching queen activity or suspected nest activity (e.g., queen disappearing into a hole or debris, exhibiting an orientation flight). Any potential nest sites were flagged and georeferenced for researcher confirmation. A researcher (ARL, VJM, or SRC) would then wait at the entrance for a minimum of 30 minutes to confirm if the site was a bumble bee nest. A nest would only be confirmed if workers were observed entering/exiting, a queen bumble bee was observed entering with pollen (which can be an indication of nest initiation) [7, 30] or if the nest was observed through the use of an endoscope camera or after excavating the nest.

The social media campaign consisted of blog posts, a dedicated web page, email newsletters, and posts to Facebook (relevant naturalist and native pollinator groups), Twitter and Instagram. The campaign included a description of where to look for nests and common indicators of nesting activity. The submission of nest sightings was encouraged, through a dedicated webform or to the Bumble Bee Watch community science program. We also monitored Bumble Bee Watch separately for any uploaded nest sightings in southern Ontario.

### On site and in-situ training with the dog-handler teams

Once the dog teams arrived in Ontario, on site and *in-situ* training was carried out from June 24-July 5, 2019. The weather conditions and start time for training sessions are shown in S1 Table. This date range was selected as it generally overlaps with the colony cycles for most bumble bee species found in southern Ontario [35]. New nest training materials were provided in Ontario (Table 1), to supplement prior dog training and to maintain both continuity and momentum, since no naturally-occurring nests had yet been confirmed when the dog teams first arrived. These additional samples (2 g) were from two commercial colonies of *Bombus impatiens*, purchased from BioBest (https://www.biobestgroup.com/), and were housed in a laboratory at York University (Toronto, Ontario). These nest training materials were stored in Ziploc^®^ bags and were either kept in a freezer or in a cooler with ice packs when used in training.

On two separate days, training was conducted at Silver Creek Conservation Area and a residential area approximately 2.5 km away. Freshly thawed training samples were placed on the surface of the ground, under vegetation/debris, or buried to a depth of approximately 5 cm using a designated hand trowel. In each case, samples were left in place for approximately 30 minutes to 1 hour to ensure enough scent dispersal before the dogs were exposed to them. An equal number of control scent areas were created using the same methods described above, using a separate hand trowel when mimicking the burial process, but without placing a nest sample. Controls ensured that the dogs would locate the bumble bee nest scent rather than cueing in on human or other non-target scents arising from sample handling and disturbed ground. The detection distance, nest ID, and vegetation surrounding the training samples (if applicable) are summarized in Table 2.

**Table 2:** Detection distances observed for human-placed material and naturally-occurring bumble bee (*Bombus* spp.) nests.

Date	Species	Human-placed or naturally-occurring?	Observed detection distance range (m)*	Site description
**June 24, 2019**	*B*. *impatiens*	Human-placed on surface	0–2 [0.5–0.75]	Georgetown, Ontario, suburban residential area, lawns/gardens
**June 25, 2019**	*B*. *impatiens*	Human-placed buried (max depth 5 cm)	0–5 [0.25]	Silver Creek Conservation Area, deciduous forest, densely vegetation understory, downed trees
**June 26, 2019**	*B*. *impatiens*	Human-placed buried (max depth 5 cm)	0–2 [0.25–0.27]	Georgetown, Ontario, suburban residential area, lawns/gardens
**June 27, 2019**	*B*. *impatiens*	Human-placed on surface	0–0.25	Silver Creek Conservation Area, deciduous forest, densely vegetation understory, downed trees
**June 29, 2019**	*B*. *impatiens/ citrinus*	Naturally-occurring underground (depth ~10–15 cm)	0.61	Norwich, Ontario, suburban residence, lawn/garden
**July 2, 2019**	*B*. *bimaculatus*	Naturally-occurring underground (depth ~ 15 cm)	0.15	Guelph, Ontario, suburban residence, lawn/garden
**July 3, 2019**	*B*. *impatiens/ citrinus*	Human-placed on surface	0–0.15	Silver Creek Conservation Area, deciduous forest, rocky, many logs, moss, dense leaf litter
**July 4. 2019**	*B*. *impatiens/ citrinus*	Human-placed on surface	0–0.25	Terra Cotta Conservation Area, open deciduous forest, few logs, dense leaf litter
**July 5, 2019**	*B*. *impatiens/ citrinus*	Human-placed on surface	0–0.15	Terra Cotta Conservation Area, open area with straw, hay bales, dirt mounds and debris

*Square brackets for detection distances indicate the more common detection distances within the range. The survey date, whether the nests were on the surface or underground, and a description of each site is also provided.

### In-situ training and exploratory ground-truthing sites

The sites selected for survey (but where *in-situ* training and ground-truthing was ultimately carried out instead) were Silver Creek Conservation Area and Terra Cotta Conservation Area. Both conservation areas are predominantly deciduous forest, but with distinct terrain. Silver Creek Conservation Area is more variable topographically than Terra Cotta, with limestone ridges, steep cliffs, rocky boulders, and crevices. The ground vegetation is often dense with underbrush, leaf litter, downed trees, and moss, and the substrate is often rocky. Terra Cotta has a relatively more open understory of deciduous forest, with less vegetation, rocks, and downed trees than Silver Creek. The ground was often covered with leaf litter. Terra Cotta also has more open areas for recreation (such as picnic areas), and an open reclaimed campground dominated by grasses, forbs and shrubs. There is also an area with discarded straw, and mounds of dirt, rocks, and other debris from past construction activities.

## Results

### Human naturally-occurring nest searches and social media nest observations

Human survey efforts at Silver Creek and Terra Cotta Conservation Areas identified six areas with high potential nest activity (i.e., high nest-searching queen activity or where queens/workers were observed entering a potential nest site). Beginning in May, ARL searched for bumble bee nest activity for 75 hours prior to volunteer involvement. In total, 17 volunteers plus three researchers (ARL, VJM, SRC) searched for bumble nests for a combined 250.25 hours. During these surveys, the bumble bee species observed foraging or flying by were *Bombus borealis* Kirby, 1837; *B*. *bimaculatus; B*. *citrinus; B*. *griseocollis* DeGeer, 1773; *B*. *impatiens; B*. *perplexus* Cresson, 1863; *B*. *rufocinctus* Cresson, 1863; and *B*. *vagans* Smith, 1854. Bumble bees were identified according to Williams et al. [35]. All six areas with presumed high probability of containing a nest were observed intensely by volunteers and bumble bee experts for a minimum of 30–90 minutes but no nests were confirmed. It is thought that the late, cool, and rainy spring experienced in southern Ontario (S2 Table) caused a corresponding delay in the emergence of queens, and they continued to be observed nest searching until June, with very few workers present in the area, suggesting few colonies were established at this time. This significantly limited our ability to locate bumble bee nests. We were unsuccessful in finding/confirming any established naturally-occurring nests via human nest searches prior to the arrival of the dog teams in southern Ontario to facilitate the transition to *in-situ* training (which would have ensured immediate availability of promptly rewardable nests for the dogs).

There were 14 respondents to our social media campaigns (excluding submissions to Bumble Bee Watch). Of these, six submitted nest locations outside of southern Ontario or that were not bumble bee nests (in one case, the nest of a carpenter bee, *Xylocopa virginica*). Of the remaining eight respondents, we were able to confirm that three had indeed sighted bumble bee nests, two of which were deemed suitable for training purposes. The other five respondents were excluded either due to limitations on gaining access to their private residence, or because we could not confirm the presence of a nest during a site visit. The rationale for excluding one of the three confirmed nests from training is explained below.

### In-situ training with the dog-handler teams

During their initial introduction in controlled settings in Montana, each of the three dogs received 126 exposures to human-placed bumble bee nest material, which they readily recognized (i.e., change of behaviour from up to 15 m away, moving towards the sample and alerting) by the end of this training period. In Ontario, they subsequently were each provided with 18 exposures to human-placed samples in residential and conservation areas (concealed or buried at a maximum depth of 5 cm), at which point, observed detection distances were narrower (< 1 m; Table 2). Note that the field-testing component described in Waters et al. [20] and O’Connor et al. [21] comprised exposure to 20 nest samples buried at depth of 10 cm. As a pre-fielding, interim training step (as described above), significant effort was invested in locating naturally-occurring nests as a way for handlers to both reinforce and confirm the ability of these individual dogs to find, and generalize to, naturally-occurring nests prior to fielding. Here, we further expand upon the process of exposing the dogs to two naturally-occurring nests, and their responses. Note the GPS coordinates for the three nests are for the city/town centers and not the location of sampling to protect the residents’ privacy.

#### Naturally-occurring nest #1 (*B*. *impatiens* with presence of cuckoo parasite, *B*. *citrinus*)

The first naturally-occurring nest, located at a resident’s property within the small rural town of Norwich, Ontario (42.983333, -80.6) (Table 2), was not immediately recognized by any of the dogs. The entrance to the nest was located within the step of a patio made of flag stones, gravel, landscape fabric and railroad ties (i.e., long, chemically treated wooden beams). A few *B*. *impatiens* workers were observed approximately every 10 minutes entering and leaving through a gap above one of the railroad ties. After excavating the nest by lifting the flag stones, removing some gravel and landscaping fabric, and providing reinforcement, all three dogs were able to independently find and then alert to the nest. Reinforcement involved verbally encouraging the dogs as they investigated the area, presenting places to sniff and finally, immediately delivering a toy to them when they got their noses over the exposed nest material. Once the nest material was exposed, a distance of 0.61 m could be measured from the observed entrance (from which bees were seen entering and exiting, and the closest point the dogs could have gotten their noses to it) relative to where the nest material itself was actually situated. Excavation of the nest (~ 10–15 cm deep) revealed approximately 30 brood cells intact and 2 opened; two dead *B*. *impatiens* queens and one dead *B*. *citrinus* female, plus one live *B*. *citrinus* female, and one live *B*. *impatiens* queen. Approximately eight *B*. *impatiens* workers were collected from the nest or as they returned to the nest. This nest (comprising approximately 1.5–2 g of material) was removed for further training purposes.

#### Naturally-occurring nest #2 (*B*. *bimaculatus*)

Two of the three dogs showed immediate recognition to this naturally-occurring nest. Reported in a suburban backyard approximately 1.7 km northwest of downtown in the city of Guelph, Ontario (43.55, -80.25), the nest was adjacent to a water feature/mini artificial pond (~ 0.4 m^2^). The nest was underground within a raised soil bed, enclosed on one side with wooden slats next to the water feature with the entrance concealed by dense vegetation. Worker bees were seen very frequently entering and leaving the nest (one every 2–5 minutes). After the initial exposure of the dogs to the nest area, all bees (approximately 150, mainly workers with a few gynes and males) were either netted as they exited the nest or as they returned to the nest area. Once the bees were removed the dogs searched the area by the nest. As described above for nest #1, this nest was similarly reinforced to the dogs, after which they all detected the nest scent within 0.15 m from the nest entrance (measured post nest excavation) when brought back around to the area.

#### Naturally-occurring nest # 3 (*B*. *bimaculatus*)

The third nest was within the foundation of a house approximately 4.3 km northwest of Georgetown, Ontario (43.646944, -79.91) and only accessible through an opening that was both elevated from the ground (~80 cm) and angled at approximately 45°. This limited air flow and potential nest scent availability for the dogs. Additionally, although bees could be seen entering and exiting one area, the entrance to, and location of the nest was difficult to pinpoint, preventing the trainer-handlers from being certain they would in fact be reinforcing and rewarding the dogs to scent emanating from that nest. Due to these factors, this nest was determined not to be suitable for dog training and we opted not to use it.

Ultimately, we did not move ahead to surveying because we sought to offer exposure to naturally-occurring nests as the transitional training step, and the WD4C trainer-handlers concluded that nearing the end of the period allocated to them, the dogs were not yet field-ready.

### Detection distances and search strategy

The detection distances observed during controlled area searches (i.e., for samples placed on the surface of the ground or buried/concealed within vegetation to a maximum depth of 5 cm) are summarized in Table 2. Detection distances to naturally-occurring nests were much shorter (less than 1 m) than training samples. At our study’s conclusion, we followed up with authors of the prior nest detection work in order to compare findings during training, testing and fielding. Previously observed detection distances spanned up to a couple of meters (S. O’Connor, pers. comm. 2019). Locating a target that, for various reasons, emits a faint scent within its natural environment requires an intensive “detail search”. This is conducted with dogs on leash to maintain slow movement and coverage of an area, their noses kept close to the ground, and with handlers repeatedly pointing out appropriate habitat features (in this case, downed logs, the base of trees, leaf debris) to search. Ensuring that dogs have ample opportunities to access important features within a given landscape type takes a long time relative to the amount of area that can be covered. The use of this search strategy relative to small scent targets (e.g., blunt-nosed leopard lizard (*Gambelia sila*) scat) is further described in Statham et al. [36] and Filazzola et al. [37]. The need for detailed searching in relation to bumble bee nests, and the landscape within which they may occur, was also encountered in the prior bumble bee nest search and detection work. S. O’Connor (pers. comm. 2019) revealed that the dog in the Waters et al. [20] study worked on leash and, although the dog in the O’Connor et al. [21] study worked off-leash, the handler did direct the dog to search certain areas by pointing to the ground or to habitat features. It was also learned that the dog successfully located nests previously identified by humans, but also at times was unable to find a nest that had been recognized or missed a known entrance (where bees had just been seen leaving) several times after sniffing it directly (S. O’Connor, pers. comm. 2019). Detailed searches for small scent targets are mentally exerting for dogs, which in turn requires pacing on the handler’s part, and remaining vigilant for signs of fatigue in the dog. After observing the behaviour of each individual dog during searches, the WD4C trainer-handlers estimated that each dog could search for no more than 20 minutes (consecutively) before requiring a break, during which time a different dog could be brought out to resume searching. A similar result was reported by O’Connor et al. [21], where 25-minute search sessions were conducted. Once a dog was given a break, they were able to continue searching for another one or two sessions, but for increasingly shorter increments.

### Opportunistic ground-truthing with the dog-handler teams

In the absence of confirmed naturally-occurring nests for training, ground truthing with the dog-handler teams was conducted within six of the target areas identified by human surveys. Field exercises were devised by the trainer-handlers, who are also seasoned field biologists, to gather complementary information around the feasibility of surveying for bumble bee nests and addressing the knowledge gap of which North American habitats may be most conducive to detailed searching by detection dogs.

On June 27, three of these targeted areas were visited at Silver Creek Conservation Area. The first was a relatively open area with many downed trees, the second was another relatively open site with dense vegetation litter and a drain pipe, and the third was a heavily vegetated location where a queen was observed to disappear (potentially indicating a nest site) and workers had been seen in the area. One of the dogs showed pronounced interest at a drainpipe and at an animal burrow, at the second and third ground truthing sites, respectively. Researchers (VJM and ARL) observed the drainpipe (60 minutes) and animal burrows (90 minutes) and inspected the two areas with endoscope cameras but could not confirm the presence of a bumble bee nest. On July 3, the dog teams visited another area at Silver Creek Conservation Area with moderately dense vegetation, many downed trees, moss, and rocky terrain. The difficult and dense terrain made it challenging for dogs and handlers to move together through the area. None of the dogs showed interest at this location. On July 4 and 5, the dogs were brought to three separate parts of Terra Cotta Conservation Area. On July 4, search grids (3 x 3 m) were conducted in an open, sloped area with downed logs and an abundance of leaf debris at one of the targeted locations. On July 5, an area with straw bales and discarded landscaping materials (soil, gravel, tree branches) was visited by the dog teams as well as an open grassy area with some dirt mounds and downed utility poles. Previous studies have indicated that bumble bees nest in straw/hay and in rock piles [e.g., 38, 39] so these seemed like appropriate/promising areas to explore. No nests were detected at any of these search locations.

## Discussion

Locating wild bumble bee nests is critical for ensuring adequate protection of bumble bee habitat and to help understand population dynamics of both rare and common species. In this study, we aimed to train detection dogs to search for bumble bee nests in southern Ontario. To build on prior work with dogs to this target, and mindful of insight gained during the course of that work [20, 21 O’Connor, pers. comm. 2019] we sought naturally-occurring nests on which to train dogs and lay a solid foundation of scent recognition and bumble bee nest/species generalization prior to surveying. We declined to proceed past the training stage to actively looking for wild nests via formal surveys (i.e., fielding) because the core part of our approach was to offer naturally-occurring nests as the transitional training step, and we did not consider that the dogs reached the point of field-readiness within the allotted timeframe. However, our experiences and observations during training may be valuable to researchers who are considering implementing this methodology within their future conservation or monitoring efforts. The three major challenges/constraints we observed are: i) prior to fielding, and given their proven ability to generalize to numerous species in prior studies, we predict dogs would likely benefit from exposure to naturally-occurring bumble bee nests; however this may neither be a realistic or feasible training scenario, certainly not as a standalone transitional training step, given the difficulty in finding naturally-occurring nests, ii) whether found by a human or by a dog, confirming a naturally-occurring find established through the presence of a resident individual in the nest is labour-intensive, time-consuming, and nuanced, and iii) study design, including selection of survey sites, must reflect the inherent realities and limitations of detection dog-handler search strategies and capabilities. Additional considerations around using detection dogs for bumble bee conservation research can be found in S1 Appendix.

### Constraint 1: Dogs might benefit from exposure to naturally-occurring bumble bee nests prior to fielding, but this may not be viable given the difficulty in finding them

Knowing that dogs can progress from finding human-handled, placed nests, to naturally-occurring nests, we sought to examine whether exposure to naturally-occurring nests could serve as a standalone transitional training step. Based on their own experiences and observations in the field of conservation detection, the trainer-handlers at WD4C have found this to be an important training steppingstone with many targets [26]. However, bumble bee nests are difficult to locate as they are often underground or in other hard to observe locations [9]. Indeed, subterranean targets offer their own inherent challenges [as discussed in 40, 41]. Finding bumble bee nests often requires large time investments and/or vast number of volunteers [i.e. 39 and demonstrated here]. Recruiting, organizing, and training volunteers (especially for conducting searches at field sites vs. backyard garden searches) can be challenging if there is not already an established volunteer network.

Home residents with access to green space may be an invaluable resource for locating bumble bee nests for the purpose of training detection dogs. They may be more likely to locate bumble bee nests within their property compared to volunteers at study sites in natural areas as they would be frequent observers of their property and are more likely to notice high bee activity in an area. Indeed, all the contacts received from community members with suspected nests were due to the residents observing a lot of bee traffic in the area. While Waters et al. [20] expressed concern that information from home resident volunteers can be biased to gardens and common species, our experience during this work shows that from a training perspective, and with the ability of dogs to generalize to other species, any increased exposure opportunities to known naturally-occurring nests would be of value. Future studies looking to identify naturally-occurring nests for the purposes of training and transitioning the dogs to naturally-occurring nests may, therefore, wish to focus their attention on home residents’ surveys of their properties. For example, Lye et al. [39] successfully located many nests using surveys directed at home residents in the UK.

One way that the involvement of detection dogs may uniquely advance bumble bee outreach and conservation efforts is in increasing public attention and engagement, potentially boosting nest site reporting. Similar excitement can also be generated in fellow researchers, resulting in unexpected opportunities for outreach, partnership, and acquisition of supplementary information (see S1 Appendix for example).

### Constraint 2: Confirming a nest find (via presence of resident bumble bees) is labour-intensive, time-consuming and nuanced

Researchers involved in prior work noted (S. O’Connor, pers. comm. 2019), and we observed, that confirming a dog has correctly located a nest and giving them a reward within a timely manner is difficult with bumble bee nests. To confirm a nest, a bee must be seen leaving or entering the nest, or the nest must be excavated. It can take a long time for a queen or workers to exit/return to a nest after foraging (e.g., minutes to hours) especially for small colonies, in cool weather, and in early spring. Similarly, nest excavation may be detrimental or counter to conservation objectives. Disrupting and altering vegetation at a nest entrance can prove disorienting to its inhabitants and in some cases could lead to nest failure (S. O’Connor, pers. comm. 2019). There can be multiple entrances to bumble bee nests [12, 13] so if a dog alerts at one entrance but the bees are using a different entrance, this might hinder accurately confirming the presence of a bumble bee nest (i.e., missing a nest that is occupied) (S. O’Connor, pers. comm. 2019). Some bumble bee species will nest above-ground in tree cavities or bird boxes [9]. To our knowledge, all training of detection dogs to locate bumble bee nests have focused on underground or surface nests. Above-ground nests in general are likely under-detected for most bumble bee nesting studies due to the difficulties in observing them [9]. Confirming nest identification is still a challenging part of this methodology that has not been well addressed particularly for underground nesting bee species but may not be as much of an issue for surface nesting species. It is important to note that previous nest detection studies have thus far focused on dogs finding (or being rewarded to) active, i.e., occupied, bumble bee nests. However, dogs can find nests at a variety of phases and activities, including failed and vacated nests (S O’Connor, pers. comm. 2019), which may also yield useful perspectives in habitat selection and preferences.

Researchers may wish to consider enlarging their survey scope accordingly and explore the viability of “proxy” targets of high conservation value. See S1 Appendix for a full discussion on the use and possibilities of proxy targets for conservation research [e.g., 44]. These proxy targets would occur in association with bumble bee nests, might be easier for dogs to locate (and be rewarded for), and could provide complementary information about nesting habitat selection and preferences. No proxy targets have yet been identified for bumble bee nests, but potential viable proxies could be considered in consultation with conservation dog professionals.

### Constraint 3a: Study designs and objectives must match the requisite search strategy using dogs

We found that the typically faint scent and concealed nature of naturally-occurring bumble bee nests require short, intensive searches. As such, it is impractical to have dog-handler teams search entire large areas for bumble bee nests. We observed this firsthand during ground-truthing exercises. Grid or subset/small-scale transect searches may be a more effective use of dogs in pre-selected locations where nest activity is suspected such as areas with: high nest-searching queen activity; previous nest sites; high traffic zones with workers (away from foraging sites); and bees performing orientation flights. Statham et al. [36] outlined important considerations around the intensive strategy used to find the diminutively scented scats of endangered blunt-nosed leopard lizards, including a search strategy which focused on shrubs that Filazzola et al. [37] determined to be positively associated with the occurrence of blunt nosed leopard lizard scats. Possible visual cues, i.e., landscape features known to be ecologically relevant to bumble bee nests, such as fence lines, hedgerows, or field margins [38, 42] can also be singled out to increase the chances of finding nests. For the aim of finding bumble bee nests using dogs, open sites without dense or tall vegetation, few downed woody debris and other litter appear to be optimal study sites [20, 21]. See S1 Appendix for suggested field site options that match these site descriptions and are known to harbour bumble bee nests.

Here, we found that the dogs could perform continuous detailed searches for about 20 minutes before they needed a break, which is similar to the 25-minute search period reported by O’Connor et al. [21]. The requirement for breaks would need to be factored into the study timeline and possibly consider increasing the number of dogs deployed to allow for more continuous searching.

### Constraint 3b: Survey timing, nesting timeframes and accurately determining presence/absence

An added challenge we experienced in our study was the unusually wet and late spring in southern Ontario in 2019 (S2 Table). In 2018, we planned to conduct *in-situ* field training and surveys in late June and early July 2019 to overlap with the phenology of many bumble bee species, assuming an “average” year [35]. The late spring meant that bumble bee workers were only just starting to be observed in the vicinity immediately before the dog teams were scheduled to arrive. This likely meant few colonies had been established and those that had were likely small in size with little wax comb (e.g., brood cells); they also would have few workers and therefore less worker traffic than if colonies were larger in size. However, it cannot be inferred from this that, correspondingly, less scent would be available for the dogs. Indeed, prior studies showed that the dog was able to detect tiny fragments of nest material left in the field [21]. Additionally, the dog in that study occasionally detected nests which were deemed to have been inactive for a period of months, and found some nest material that was very small, or almost entirely consumed by wax moths (S. O’Connor, pers. comm. 2019). In this regard, Goulson et al. [43] report on numerous observations of species (including predators) interacting with bumble bee nests, mostly destructively, thereby offering additional insight on the complexity that this target presents both to dogs and those tasked with confirming a find. Accordingly, researchers may wish to incorporate not only occupied nests but also previously occupied locations (e.g., overwintering sites used by queens) into their study design and objectives. Future studies must consider the best survey timeframe to maximize the likelihood of finding different species, and/or the most nests.

### Constraint 3c: Study sites must be selected with an understanding of the limitations that certain habitats may pose to dog-handler searches

The effectiveness of detection dogs varies according to the environment in which they operate. This has specifically proven to be the case with bumble bee nests, where prior work showed varying levels of nest detection success within several different types of habitats in which surveys were conducted [20, 21, and see Table 3]. Note especially the difference in reported find rate success and searching relative to effort in Waters et al. [20] working in open island habitat versus by O’Connor et al. [21] in certain more vegetated, challenging terrains. Thus, when pooled and reported across habitat types, dog nest find rate is misleading and should be considered accordingly (see also Table 3 notes). Bumble bees may nest in a plethora of different habitats, not all of which can readily be traversed or efficiently searched by dog-handler teams. Numerous studies have reported on the role of vegetation in find rates [see for examples 44, 45] and it was specifically described as a limiting factor in bumble bee nest detection by both Waters et al. [20] and O’Connor et al. [21]. Finally, relative to nest find rates, when human versus dog performance is compared, and where some of the same areas are being searched, it would behoove researchers to specify whether or not a dog found any of the same nests that humans found, or different nests, and the species of bee associated respectively with human versus dog finds. Researchers wishing to explore the incorporation of detection dogs for future bumble bee nest related efforts should carefully consider whether their focal location and the habitat therein would lend itself well to the use of this monitoring method.

**Table 3:** Summary of study aims, parameters, findings and outlooks from prior published bumble bee nest detection dog work.

Study	Aims	Location(s)	Timing of study	Finding(s)	Outlook:
**Waters et al. (2011)**	Train dog to find nests of rare bee species. Ultimate goal: Assessing nest density for estimate of effective population size	Hebridean Island of Tiree (Scotland) [Tiree described as having an ‘unusually high’ density of bumble bee species]	August and September (2006) Coinciding with the end of the nesting cycle	Dog find rate: 33 nests over 30 ha 4 spp. represented Recorded nest densities: *B*. *muscorum*: 1.86 nests/ha (machair) ^a^ *B*. *distinguendus*: 0.533 nests/ha (dunes) *B*. *lapidarius*: 0.267 nests/ha (machair) *B*. *jonellus*: 0.133 nests/ha, single nest find (lowland heath) Patterns of habitat preferences could be discerned according to species [Nests of *B*. *muscorum*, most abundant species, was found at highest rate]	The technique has great potential, but using a dog in dense vegetation limits the effectiveness [Testing and searches were all executed in open habitat The island of Tiree is almost entirely devoid of densely vegetated habitat]
**O’Connor et al. (2012)**	Comparison of: 1) efficiency of two detection dogs trained to bumble bee nests based on the performance of the dog in the current study and those study conditions relative to that of the dog in Waters et al. (2011) 2) ability of a dog to locate nests when carrying out repeat searches of agricultural habitats through the season 3) efficiency of a dog compared with human volunteers at finding nests in woodland	Rural and woodland habitats in the United Kingdom: Farmland near Stirling, Scotland hedgerow fence line bank (i.e., steeply sloping earth bordering lanes and ditches) long grass (>15 cm), short grass (<10 cm) woodland edge (within 10 m of) Open woodland habitat	May to August (2008)	Dog find rate: In rural habitats: 9 nests of 4 species *B*. *terrestris* (n = 3) *B*. *pascuorum* (n = 3) *B*. *lucorum* (n = 2) *B*. *hortorum* (n = 1) Overall rate: 1 nest/19 h 24 min search time However, on a bee nest per habitat basis: 3 nests in woodland edge habitat 3 nests within hedgerows 1 nest in short grass 1 nest in long grass 1 nest in bank habitats 0 nests detected along fences Free searches—human volunteers vs dog in woodland Similar find rates = 1 nest / 1 h 20 min Fixed search by humans in woodland 4 nests found by volunteers 3 x *B*. *terrestris* + 1 x *B*. *pratorum*). 1 nest for 3 h 20 min of searching Free search by humans in woodland 10 nests found by volunteers 7 x *B*. *terrestris* + 2 x *B*. *lucorum* + 1 x *B*. *pratorum* 1 nest for 1 h 20 min The dog located 10 nests during woodland searches of the same area as the volunteers 7 x *B*. *terrestris* + 1 *x B*. *lucorum* + 1x *B*. *hortorum* + 1 x *B*. *lapidarius*	Detection dogs are not a cost-effective method for locating bumble bee nests, especially relative to volunteers Fixed searches are appropriate for an aim of estimating bumble bee nest density Free searches are a better method for finding many nests Novice volunteers performed as well as experienced ones
**Goulson et al. (2018)**	Find naturally-occurring nests to install cameras and record activity, detect gyne production, and record visits by vertebrate predators Screened workers for internal parasites, providing a detailed account of the factors affecting the fates of 47 bumblebee nests.	See O’Connor et al. (2012)	See O’Connor et al. (2012)	47 naturally-occurring nests found by a detection dog^b^ *and* volunteers. This study made use of nests found during work conducted by, and described in, O’Connor et al. (2012)	Peripheral/opportunistic use of already trained detection dog, i.e., no specific conclusions relative to their efficacy. But worth highlighting the detection dog tangibly contributed in this way to bumble bee nest detection efforts and conservation/research efforts].

Text in square brackets denote author notes from the study.

^a^ rare habitat confined to west Scotland and Ireland, consists of flat coastal plain of species-rich grassland growing on wind-blown shell sand.

^b^ same dog that participated in O’Connor et al. (2012).

Waters J, O’Connor S, Park KJ, Goulson D. Testing a detection dog to locate bumblebee colonies and estimate nest density. Apidologie. 2011 Mar;42(2):200–5.

O’Connor S, Park KJ, Goulson D. Humans versus dogs; a comparison of methods for the detection of bumble bee nests. J Apic Res. 2012;51(2):204–11.

Goulson D, O’Connor S, Park KJ. The impacts of predators and parasites on wild bumblebee colonies. Ecol Entomol. 2018;43(2):168–81.

## Conclusion

Detection dogs can find bumble bee nests in the wild [20, 21]. However, it appears that as a monitoring tool, using detection dogs as a method of locating bumble bee nests for ecological studies has limited applications with significant drawbacks to its implementation, especially regarding nest confirmation and timely reward. Some of these constraints could perhaps be mitigated to a certain degree, with careful considerations of study question and design. This includes developing research questions that can feasibly be answered with this method, designing studies around directed and detailed searches, locating naturally-occurring bumble bee nests for training dogs prior to their deployment, choosing study sites that are conducive to dog search strategy and capabilities, determining how nest confirmation will occur, and potentially incorporating inactive/former bee nests (versus, or in addition to, occupied ones). It may be possible to pair some modeling and projection with a selection of small subplots to detail search within a larger area. Additionally, the ability of dogs to generalize across bumble bee species, and the potential greater facility in finding proxy targets important to bumble bees, remains worthy of consideration in exploring the future viability of any applications.

The exposure of dogs in training to naturally-occurring nests *in-situ*, the inherent olfactory challenges posed by nests and the nuances and difficulties posed to people for confirmation purposes all make this a difficult target on which to train and field dogs. Bearing in mind these now well-established limitations of using detection dogs to further bumble bee research and conservation objectives, at least in terms of seeking naturally-occurring nests in certain types of habitats, further exploratory work is nonetheless warranted to explore the potential ways that the involvement of detection dogs could uniquely help researchers gain insight into bumble bee habitat selection, occupancy or nesting preference.

## Supporting information

S1 Table Detection dog session parameters during on-site and *in-situ* training in Ontario, Canada (June 24 –July 5, 2019). Summary of work session parameters of the dog-handler teams, which depended on the prevailing weather conditions, to capitalize on cooler temperatures and reduce heat stress opportunities on the dogs. The session timeframes and temperatures also coincided with reduced bumble bee activity to minimize dogs coming into contact with bees.
(DOCX)

S2 Table A comparison of historical average (1981–2010) and 2019 temperature and precipitation data for a weather station located in Georgetown, Ontario. (Georgetown WWTP ID = 6152695, 43.640005, -79.879172, ~8 km away from Silver Creek Conservation Area, https://climate.weather.gc.ca/).
(DOCX)

S1 Appendix Additional considerations for using detection dogs to find bumble bee nests and in related conservation efforts. (DOCX)
