# Supplementary material for: Training and usage of detection dogs to better understand bumble bee nesting habitat: Challenges and opportunities
Source: PLoS One. 2021 May 12;16(5):e0249248. doi: 10.1371/journal.pone.0249248 (PMC8115777; doi:10.1371/journal.pone.0249248)
Supplement: S1 Table — Summary of work session parameters of the dog-handler teams, which depended on the prevailing weather conditions, to capitalize on cooler temperatures and reduce heat stress opportunities on the dogs. The session timeframes and temperatures also coincided with reduced bumble bee activity to minimize dogs coming into contact with bees. (DOCX) [file pone.0249248.s001.docx]

**S1 Table.** Detection dog session parameters during on-site and *in-situ* training in Ontario, Canada (June 24 – July 5, 2019)

| Start times | 6 – 7 am |
| --- | --- |
| Starting temperatures | 11 – 17.2°C |
| End times | 10 am – 1 pm |
| Ending temperatures | 24 – 27.8°C |

Summary of work session parameters of the dog-handler teams, which depended on the prevailing weather conditions, to capitalize on cooler temperatures and reduce heat stress opportunities on the dogs. The session timeframes and temperatures also coincided with reduced bumble bee activity to minimize dogs coming into contact with bees.
