# Supplementary material for: Training and usage of detection dogs to better understand bumble bee nesting habitat: Challenges and opportunities
Source: PLoS One. 2021 May 12;16(5):e0249248. doi: 10.1371/journal.pone.0249248 (PMC8115777; doi:10.1371/journal.pone.0249248)
Supplement: S2 Table — (Georgetown WWTP ID = 6152695, 43.640005, -79.879172, ~8 km away from Silver Creek Conservation Area, https://climate.weather.gc.ca/). (DOCX) [file pone.0249248.s002.docx]

**S2 Table: A comparison of historical average (1981-2010) and 2019 temperature and precipitation data for a weather station located in Georgetown, Ontario**

| Month | Temperature daily average 1981-2010 (°C) | Temperature daily average 2019 (°C) | Precipitation average 1981-2010 (mm) | Precipitation average 2019 (mm) |
| --- | --- | --- | --- | --- |
| April | 6.0 | 4.8 | 76.5 | 93.4 |
| May | 12.3 | 11.2 | 79.3 | 97.6 |
| June | 17.4 | 17.4 | 74.8 | 108.6 |

(Georgetown WWTP ID= 6152695, 43.640005, -79.879172, ~8 km away from Silver Creek Conservation Area, https://climate.weather.gc.ca/).
