# Supplementary material for: Training and usage of detection dogs to better understand bumble bee nesting habitat: Challenges and opportunities
Source: PLoS One. 2021 May 12;16(5):e0249248. doi: 10.1371/journal.pone.0249248 (PMC8115777; doi:10.1371/journal.pone.0249248)
Supplement: S1 Appendix — (DOCX) [file pone.0249248.s003.docx]

**S1 Appendix: Additional considerations for using detection dogs to find bumble bee nests and in related conservation efforts**

# Types of dogs used in conservation detection applications

In this study, the dogs that participated were carefully vetted and specifically selected for possessing a suite of characteristics including obsession with toys and desire to work with a handler. These traits were considered independently of breed, though candidate dogs are often of working breeds. A detailed discussion of conservation detection relative to this training method, and of the types of dogs sought under this approach, can be found in Mackay et al. [1] and Hurt and Smith [2].

# Bumble bee nest training considerations

As in prior work, this paper outlines the challenges that finding naturally-occurring bumble bee nests pose for people. For example, we found that volunteers often flagged potential nest sites, but after bumble bee researchers observed the location and discussed the volunteer’s observations, we discovered that most of these potential nests were locations of interest for nest-searching queens. We suspected this might have been due to the varying behaviour observed between different individual nest-searching queens where some move quickly and do not spend a great deal of time investigating aspects of the environment while others could spend upwards of 30 minutes inspecting a single feature.

Given the difficulty in procuring wild nest samples, and the ability of dogs to generalize (as described in our paper), captive-reared nest material from a research laboratory or commercial sources can be used for training. Note that the two dogs that participated in the Waters et al. [3] and the O’Connor et al. [4] studies were initially trained and partially field-tested using commercially reared nests. However, we emphasize that if, at the field-testing stage, an opportunity for giving dogs exposure to known wild nests should arise, it could be formative both for the dogs and their handlers. Any additional exposure to naturally-occurring nests also helps further refine the dog handler’s search image, which only assists them in better directing the dog during detailed searches.

# Suggested field sites for deploying detection dogs to locate bumble bee nests

Field site options that are known to have bumble bee nests include open deciduous forests, short grass meadows, agricultural fields and field margins, sand dunes, and open shrubland [9]. Similarly, bumble bees have been known to nest in distinct features and landmarks such as farm (out)buildings, discarded machinery and old vehicles, and around drainage ditches (E. Venturini, pers. comm. 2020): these represent potentially more readily accessible and less ambiguous search areas for the dogs. As such, outreach to owners of properties (including farms) where outbuildings, greenhouses, old machinery and derelict vehicles abound could also be specifically targeted as places offering bumble bee nest exposure opportunities for dogs.

# Proxy targets for bumble bee nests

Scent targets that are associated with the study species, i.e., proxy targets, have been used in previous conservation research involving detection dogs. For example, dogs were trained to threatened Kincaid’s lupine (*Lupinus sulphureus* ssp. *Kincaidii*), a primary host plant to the endangered Fender’s blue butterfly (*Icaricia icarioides fenderi*), thereby allowing information about habitat quality to be gathered without having to train the dogs to the scent of the rare butterfly itself [8]. No proxy targets have yet been identified for bumble bee nests. Potential proxy targets can be considered in consultation with conservation dog professionals to determine their viability from a detection dog perspective, and, if viable, to estimate the value of these targets relative to the study objectives and develop appropriate methodologies accordingly.

# The involvement of detection dogs may facilitate future research collaborations

Members of the public are often engaged by research involving detection dogs. For example, the residents we visited during our study were very excited by the participation of the dogs, which we also suspect generated an increased response via social media. This may also come to the attention of researchers, generating possibilities for collaboration that might not otherwise have arisen. For example, early discussions were initiated with various pollinator experts by the WD4C trainer-handlers to procure nest sampling material for dog training. These discussions revealed that a group in Central Illinois (USA) were studying house wrens and have 700 nest boxes (about 1.5m above the ground) in a woodland and savannah habitat [5], and it has been reported that between eight and twelve have been occupied by bumble bees annually over the past three years (B. M. Sadd, pers. comm, 2020). With this in mind, a call could be put out to researchers using nest boxes as a study tool, with boxes occupied by bumble bees potentially lowered to ground level, thus serving as an interim training tool for dogs, and providing supplementary information about habitat occupancy gained in the process. The unique ambassador role that conservation detection dogs can play in paving the way for public engagement and deeper research collaborations is further described in Sawchuk et al. [6] and Woollett (Smith) et al. [7].

# References

1. MacKay P, Smith DA, Long RA, Parker M. Scat detection dogs. In: Long R, MacKay P, Zielinski W, Ray J, editors. Noninvasive survey methods for carnivores. Washington, D.C.: Island Press; 2008. pp. 183–222.

2. Hurt A, Smith DA. Canine ergonomics: the science of working dogs. Helton W, editor. Boca Raton, Florida, USA: CRC Press, Taylor and Francis Group; 2009.

3. Waters J, O’Connor S, Park KJ, Goulson D. Testing a detection dog to locate bumblebee colonies and estimate nest density. Apidologie. 2011;42: 200–205. doi:10.1051/apido/2010056

4. O’Connor S, Park KJ, Goulson D. Humans versus dogs; a comparison of methods for the detection of bumble bee nests. J Apic Res. 2012;51: 204–211. doi:10.3896/IBRA.1.51.2.09

5. Bowers EK, Grindstaff JL, Soukup SS, Drilling NE, Eckerle KP, Sakaluk SK, et al. Spring temperatures influence selection on breeding date and the potential for phenological mismatch in a migratory bird. Ecology. 2016;97: 2880–2891.

6. Sawchuk C. Intercepting invasive invertebrate species before they infest waterbodies: The inception and implementation of Alberta’s dedicated canine mussel inspection program. In: Richards NL, editor. Using Detection dogs to monitor aquatic ecosystem health and protect aquatic resources. Palgrave Macmillan, Cham. pp. 119-165

7. Woollett (Smith) DA, Hurt A, Richards NL. The current and future roles of free-ranging detection dogs in conservation efforts. In: Gompper ME, editor. Free ranging dogs and wildlife conservation. Oxford, UK: Oxford University Press; 2014. pp. 239–264.

8. Vesely DG. Training of conservation detection dogs to locate kincaid’s lupine (*Lupinus sulphureus ssp. kincaidii*). 2008.

9. Liczner AR, Colla SR. A systematic review of the nesting and overwintering habitat of bumble bees globally. J Insect Conserv. 2019. doi:10.1007/s10841-019-00173-7
